# Caregivers' Perception about the Relationship between Oral Health and Overall Health in Individuals with Disability in Qatif, Saudi Arabia: A Cross-Sectional Study

**DOI:** 10.1155/2022/8586882

**Published:** 2022-10-03

**Authors:** Marwa Alalshaikh, Rasha Alsheikh, Amal Alfaraj, Khalifa S. Al-Khalifa

**Affiliations:** ^1^Periodontics Fellowship Program, College of Dentistry, Imam Abdulrahman Bin Faisal University, Dammam, Saudi Arabia; ^2^Department of Restorative Dental Science, College of Dentistry, Imam Abdulrahman Bin Faisal University, Dammam, Saudi Arabia; ^3^Implant Fellow, Department of Prosthodontics, Indiana University School of Dentistry, Indianapolis, Indiana, USA; ^4^Department of Preventive Dental Sciences, College of Dentistry, Imam Abdulrahman Bin Faisal University, Dammam, Saudi Arabia

## Abstract

**Background:**

In Saudi Arabia, there are many people with disabilities that do not receive adequate healthcare, especially in the field of dentistry.

**Objective:**

This study focused on assessing caregivers' perception of the relationship between oral healthcare and the overall health status of individuals with special healthcare needs (SHCN) in Qatif, Saudi Arabia. *Methodology Design*. This cross-sectional study was conducted using a questionnaire that was divided into two sections. The first part included demographic information such as the age and gender of both the caregiver and the person with a disability and the type of disability. The second part investigated the systemic health of the individual with a disability and the caregivers' perception of the relationship between the management and treatment of systemic health and oral healthcare in relation to the overall health status. The results of descriptive analyses were summarized using frequency distribution tables. Bivariate analyses (chi-square test) were also performed. Significant differences were considered at a *p* value of 0.05. *Setting*. Initiated in school setting.

**Results:**

A total of 186 caregivers participated in the study. As much as 83.3% agree that oral health affects overall health, and 48.9% agreed that untreated dental problems could affect cardiac health. Age was the only factor found to be significantly associated with caregiver perception as younger caregivers were more likely to rightly understand the relationship between oral health and general health (*p* < 0.05).

**Conclusion:**

This study has shown the extent to which caregivers of individuals with special care needs to understand the role of oral health in the general health of an individual. Three-quarters of the caregivers agree that dental health affects overall health, and age is a factor that might influence this understanding.

## 1. Introduction

Individuals with disabilities are the most marginalized groups in society [1, 2]. Discrimination, lack of care, and inequity are obstacles preventing them from their right to proper education and healthcare [3, 4]. In Saudi Arabia, it is estimated that 3.3% of the population has a disability, and only a small percentage of those benefit from available health services [3–5]. Patients with disabilities present with various conditions and level of impairment that frequently includes systemic complications and illnesses [1–6]. In addition, it has been reported that the prevalence and severity of dental diseases in individuals with disabilities are significantly higher than in the rest of the population [7–10]. The magnitude of the problem is even worse when thinking of patients with disabilities who already may have compromised physical health [7]. Furthermore, most parents of individuals with disabilities showed adequate knowledge regarding dental care and its impact on the individuals' oral and systemic health [11].

Individuals with physical disability are completely dependent on their caregivers to maintain good oral hygiene, and such individuals are subjected to increase consumption of sugar containing medications that contribute to higher caries and periodontal diseases risk [12]. Several studies discussed how oral health conditions are closely related to overall general health and systemic diseases [12–16]. In the case of individuals with disabilities, systemic health problems are a big concern because poor dental health may further compromise their general health [13, 14, 16, 17]. Poor dental health may also aggravate negative self-image in these individuals resulting in poor social interaction [18]. Most of these individuals have medical conditions that sometimes require the use of medication that could be sweetened which in return might cause dental caries for this high-risk group [17, 19, 20]. Inflamed periodontal tissue and carious teeth due to lack of proper oral hygiene are linked to cardiovascular diseases and bacterial endocarditis in patients with cardiac defects [16]. According to several studies, the main reason for morbidity in disabled individuals is the exacerbation of respiratory infections and chronic health problems such as heart disease, lung disorders, arthritis, and type II diabetes; aging-related health problems may manifest themselves 15–20 years earlier in individuals with disabilities population compared to others [12, 16, 21].

A previous study in Saudi Arabia reported that caregivers lack the knowledge and awareness about the link between general and oral health [11]. There is a considerable lack of data about oral care needs among individuals with special healthcare in Saudi Arabia, as well as the understanding of the caregivers' lack of knowledge of the importance of oral health and its impact on the overall health. The purpose of this study was to assess caregivers' perception of the relationship between oral healthcare and the overall health status in individuals with special healthcare needs (SHCN) in Qatif, Saudi Arabia.

## 2. Methodology

### 2.1. Study Population

This cross-sectional study is a part of a larger study that was conducted between February and April 2019 to investigate the oral health knowledge, barriers to access to care, and general health perception of special needs' caregivers population in Qatif city, Eastern Province of Saudi Arabia, with a population of about 524,400 [22, 23]. Public schools with integrated special needs education, centers associated with the Ministry of Social Affairs, as well as charity organizations providing support for the care of individuals with special healthcare needs in Qatif were approached for the study. The sample size was calculated considering a 90% confidence level, 5% margin of error, and a 50% response distribution using the sample size calculator (https://www.raosoft.com/samplesize.html). The expected population of special needs in Qatif area was 500 according to the latest statistics from the Ministry of Social Affairs. The minimum required sample size was calculated to be 176.

Eligible participants were selected using a convenience sampling technique where willing participants were sequentially recruited till the required sample had been gotten. Each participant received a detailed description of the study and was requested to provide informed consent. The recruitment of the participants was based on the following inclusion/exclusion criteria:

#### 2.1.1. Inclusion and Exclusion Criteria

Participants must be parents or caregivers of individuals with special healthcare needs individuals, at least 18 years or older, and who can fully understand the contents of the survey and able to provide informed consent were included in the study. While participants who had not been taking care of an individual with special healthcare needs as well as incomplete information on the survey were excluded from the study.

### 2.2. Data Collection

All eligible caregivers across the eleven selected study locations were asked to join the study and informed about their prerogative to decide whether to participate and/or withdraw at any time. Ethical approval was obtained from the Institutional Review Board at the Imam Abdulrahman bin Faisal University, Dammam (EA: 2014040).

A modified questionnaire from previous studies evaluating caregivers' perception of the relationship of oral healthcare with overall health status was developed in Arabic and subsequently translated back into English language [11, 24–26]. The questionnaire was pilot tested on 10 caregivers, who were not later included in the study. The survey was distributed and read by random people to evaluate whether the questions effectively capture the topic under investigation; then, it was checked by an expert on questionnaire construction. After collecting the papers, the statistics ran to test their responses and any irrelevant question dropped by the investigator. The questionnaire was divided into two sections. The first part included demographic information such as the age and gender of both the caregiver and the person with disability and the type of disability. The second part investigated the systemic health of the individual with disability and the caregivers' perception about the relationship between the management and treatment of systemic health and oral healthcare in relation to the overall health status.

Data were collected, entered, and organized using the Statistical Package for Social Science (SPSS) software, version 22 (Chicago, IL, USA). The results of descriptive analyses were summarized using frequency distribution tables. Bivariate analyses (chi-square test) were also performed to assess any association between the study variables. Significant differences were considered at a *p* value of 0.05.

## 3. Results

A total of 186 participants across the 11 special needs centers and schools in Qatif responded to the survey. Of this population, majority were females (64.5%), while male caregivers accounted for 35.5%. Caregivers with ages under 25 years constituted 21.0% of the participants, while 28.0% were within the 25–34 years age range, 30.6% within the 35–44 years age range, and 20.4% older than 45 years. Caregivers with education below high school were 29.6%, while those with high school education and education at the college level and higher were 36.0% and 34.4%, respectively. Most of the individuals with special healthcare needs being cared for by the caregivers had developmental disabilities (44.6%), while the reminder had either sensory impairment (26.9%), physical disability (18.3%), or behavioral/emotional disorder (10.2%) (Table 1).

Based on the responses of the caregivers (Table 2), a little over half (50.5%) of the individuals with disability have health issues. Approximately one out of five of the SHCN individuals were suffering from a heart condition (22.5%). Hypertension was the second most common disease associated with disability (14.9%). This was followed by diabetes, bleeding disorder, asthma, and allergy (12.8%, 12.8%, 11.7%, and 11.7%), respectively. Anaemia/sickle cell anaemia was reported in 9.6% of the SHCN individuals; 6.4% suffered from renal conditions and 4.3% had liver disease. At the time of data collection, more than half (66.0%) of patients were taking medications for their condition.

When asked questions, surrounding their knowledge of the relationship between oral health and health in general, most of the caregivers (83.3%) indicated that they aware of the effects of dental health on the overall health. More than half (62.9%) of the caregivers understood the relationship between the poor oral health and serious systemic health issues. As much as 48.9% of the caregivers were fully aware of the consequences of poor dental health and poorly managed dental conditions on the heart. Interestingly, 87.6% of them found that it is important to inform their ward's dentist about past and current medical history including allergies, previous treatments, and usage of medications before starting any treatment. Approximately three out of four caregivers (78.0%) agreed that some dental procedures may need to be approved by the patient's physician before they are performed; while more than half (57.0%) agreed that some medications cause harm to the oral cavity such as dental caries and periodontal diseases. About 60.2% of the caregivers understood that some individuals with disabilities are at a higher risk of systemic conditions associated with poor oral health. When asked if communication between the dental team and the health provider is important and will offer the best care for the patients, 87% of them answered in the affirmative (Table 2).

The study went ahead to assess relationships between caregiver demographics (caregivers' gender, education, and age) and their perception about the relationship between the management and treatment of systemic health and oral healthcare in relation to the overall health status (Table 3). Caregiver's age was found to be significantly associated with acknowledging the need to inform the dentist about medical condition (*p* < 0.05). More specifically, 94.5% of caregivers younger than 34 years agreed that is important to provide the dentist with the past/current medical history as opposed to 81.1% of those 35 years and older who did. None of the other associations between caregiver perceptions about the relationship between oral and general health and caregiver demographics were found to be statistically significant.

## 4. Discussion

The present study has provided information about the caregivers' perception and understanding of the relation between oral healthcare and the general health status in individuals with SHCN in Qatif, Saudi Arabia. People with SHCN often have unmet health and dental care needs despite the fact of being at considerable risk of poor oral hygiene and oral diseases [27–30]. In this study, a considerable proportion of the SHCN individuals had health issues ranging from a heart condition to diabetes, asthma, allergies, and renal conditions. All the listed conditions as experienced by the SHCN individuals in this study and medications prescribed to manage have been found to be associated with a negative impact on oral health [31, 32]. Periodontal disease, xerostomia, and considerable risk of caries are some of the related oral manifestations of these conditions [31–33]. These oral conditions in return have also been documented to have a negative impact on the overall health status of the individual [31–38].

With the limited access to dental care, it is the responsibility of the caregivers to maintain good oral hygiene for the SHCN individuals. The awareness of the caregivers participating in this study was mostly satisfactory in relation to the link between good dental health and general health, which agrees with findings in other published studies [39, 40]. However, only half agreed that poor dental health could affect heart condition if not treated, while a little above one-third of the participants had no knowledge about the relationship. This shows a disconnection in the knowledge of the caregivers; although they know that oral health is essential, they are still not sure or deny any link between it and the heart condition. It is more apparent with the follow-up question that a larger proportion agreed with that some individuals with SHCN are at a higher risk of health problems related to oral health.

On the positive side, the agreement was higher when asked if the dentist should be aware of the individual current/past health condition (88%), if dental procedures should be approved by the physician (78.5%), and whether the dentist should be in contact with other health providers to offer the best treatment (87%). Despite the disconnection in the caregivers' knowledge, they are aware of the dentist's role and the importance of having the dentist in line with all other health providers, and this is the positive attitude desired from the personnel in charge of taking care of such unprivileged individuals. The information gathered in this study would help in assessing the caregivers' knowledge level, lacking links, and consequently, what kind of intervention is needed [41].

In this study, the larger proportion of the caregivers was females (64.5%). While other studies have suggested that females behave better concerning oral healthcare measures, the findings in this study show no difference between both genders in this regard [42, 43]. The same finding for the caregivers' education level where it was expected that there will be more desirable behavior among participants with higher education, but there was no detected difference. Educational level is continuously used to identify specific knowledge and behavior with higher educational levels persistently correlating with better behavior and understanding of the oral hygiene impact on dental health and, consequently, the general health status [44, 45]. The results showed no difference in the participant behavior among different educational levels despite that they were divided almost equally between under high school (29.6%), high school (36.0%), and college or higher education (34.4%). This indicates that their knowledge and attitude are not influenced by their formal education, but by their practice or other exposure to the information resource, such as health awareness campaigns, lectures, the Internet, or other sources [45].

There was a significant correlation between the caregivers' response and their age group (*p* > 0.05). More than half (52.8%) of the caregivers who strongly agreed to the importance of informing the dentist about the individuals with disabilities past/present health conditions, allergies, treatment, or usage of medication were between the age of 25–44 years, and more than half the participants agreed or strongly agreed that some individual with SHCN are at a higher risk of health problems related to oral health were between the age of 35–44 years. This can be due to the incapability of the older caregivers to understand the questionnaire, the methods used to raise the caregivers' awareness, or the lack of exposure of the younger aged caregivers to such methods. Years of work experience can also be a factor as those who have long years of experience gain confidence in the familiar ways they used, as well as the experience they gained which render them comfortable and resistance to change by resisting new information or instruction. The same for the younger caregivers as fewer years of experience might be the reason for not being exposed to such knowledge, and the lack of professional education can be an added factor to explain such results [46].

While this study took care to address potential limitations, it is important to consider the role those unavoidable limitations may pose in generalizing the findings of this study. Study data were collected using self-reported questionnaires which might be considered a limitation, as there is an increased risk of bias in cases where some participants decide to answer questions based on social desirability rather than objective reasoning. Convenient sampling as well as the focus in a certain region can be added to the study limitations. Future studies may consider alternative data collection approaches to avoid this bias. However, the study was able to establish participants perceptions concerning the role of oral health in overall health. Also, the results reflect a positive knowledge level among the responding caregivers, which theoretically will reflect in a better attitude toward the SHCN individual's dental hygiene methods, and subsequently prevent dental problems and promote the dental and general health of special needs SHCN individuals under their care.

In Saudi Arabia, reported disability reference is estimated to be as high as 3.3% of the total population which reflects a high prevalence [47]. In the light of the findings of this study, targeted efforts should be made to ensure the optimal dental care of such vulnerable individuals. It is stated that the knowledge of the caregivers of the importance of dental health reflects positively on the individuals they care for [42]. To increase the productivity and influence of any designed intervention, it is essential to know the level of the caregivers' knowledge and the lost links.

In order to increase general knowledge of the link between oral health and overall health, targeted educational programs should be designed to improve caregivers' knowledge further and fill existing gaps. Further investigation to link the knowledge level and the actual dental condition of the SHCN is recommended. The dentist should be considered as an essential member of the healthcare team to help promote the oral and general health of SHCN individuals. A referral channel should also be established between dental and medical providers to address any detected oral manifestation and oral diseases.

## 5. Conclusion

This study has shown the extent to which caregivers of individuals with special care needs to understand the role of oral health in the general health of an individual. Approximately three-quarters agree that dental health affects overall health, and age is a factor that might influence this understanding.

## Figures and Tables

**Table 1:** Demographic distribution of study participants.

Group	Variables	Values (*n* = 186)	*N*	%
Caregivers	Gender	Male	66	35.5
		Female	120	64.5
	Age	12–34 years	91	48.92
		>35 years	95	51.07
	Level of education	<high school/no education	55	29.6
		High school and above	131	70.43

Special needs individuals	Special needs types	Behavioral and emotional	19	10.2
		Developmental disability	83	44.6
		Physically disabled/others	34	18.3
		Sensory impairment	50	26.9
	Gender	Male	125	67.2
		Female	61	32.8

**Table 2:** Perception of caregivers about the relationship between oral health and overall health.

Variables	Values	*N*	%
Health condition status			
Does the person you care for suffer from any health condition?	Yes	94	50.5
	No	87	46.8
	Do not know	5	2.7
Type of health condition	Heart condition	21	22.3
	Asthma	11	11.7
	Thalassemia	3	3.2
	Diabetes	12	12.8
	Immunodeficiency	3	3.2
	Liver disease	4	4.3
	Kidney diseases	6	6.4
	Anaemia/sickle cell anaemia	9	9.6
	Bleeding disorder	12	12.8
	Cancer	3	3.2
	Allergy	11	11.7
	Hypertension	14	14.9
	Others	8	8.5
Is the person you care for on any medication? (*n* = 94)	Yes	62	66.0
	No	30	31.9
	Do not know	2	2.1

Health giver perception			
Dental health affects overall health	Yes	155	83.3
	No	18	9.7
	Do not know	13	7.0
Poor dental health is linked to many serious diseases and conditions in the body	Yes	117	62.9
	No	26	14.0
	Do not know	43	23.1
Poor oral health and some dental procedures can affect heart health if not managed properly	Yes	91	48.9
	No	26	14.0
	Do not know	69	37.1
It is important to inform the dentist about any past/current health conditions, allergies, treatments, or usage of medications	Yes	163	87.6
	No	6	3.2
	Do not know	17	9.1
Some dental procedures need to be approved by the physician according to the patient health condition	Yes	145	78.0
	No	13	7.0
	Do not know	28	15.1
Continuous usage of some medication can cause oral health problems like tooth decay and inflamed gums	Yes	106	57.0
	No	32	17.2
	Do not know	48	25.8
Some individuals with special needs are at a higher risk of health problems related to poor oral health	Yes	112	60.2
	No	18	9.7
	Do not know	56	30.1
It is important the dentist team communicate with other health providers to offer the best treatment for special needs patients	Yes	161	86.6
	No	4	2.2
	Do not know	21	11.3

**Table 3:** The association between caregivers' age, gender, and education with perception of caregivers about the relationship between oral health and overall health.

Variables	*N*	Caregiver age			Caregiver gender			Caregiver education		
		12–34 years	≥35 years	*P*	Male	Female	*P*	< high school/no education	High school and above	*P*
Dental health affects overall health										
Yes	155	79 (86.8%)	76 (80.0%)	0.213	54 (81.8%)	101 (84.2%)	0.681	48 (87.3%)	107 (81.7%)	0.350
No^*∗*^	31	12 (13.2%)	19 (20.0%)		12 (18.2%)	19 (15.8%)		7 (12.7%)	24 (18.3%)	

Poor dental health is linked to many serious diseases and conditions in the body										
Yes	117	56 (61.5%)	61 (64.2%)	0.706	43 (65.2%)	74 (61.7%)	0.638	32 (58.2%)	85 (64.9%)	0.388
No^*∗*^	69	35 (38.5%)	34 (35.8%)		23 (34.8%)	46 (38.3%)		23 (41.8%)	46 (35.1%)	

Poor oral health and some dental procedures can affect heart health if not managed properly										
Yes	91	39 (42.9%)	52 (54.7%)	0.105	34 (51.5%)	57 (47.5%)	0.600	26 (47.3%)	65 (49.6%)	0.770
No^*∗*^	95	52 (57.1%)	43 (45.3%)		32 (48.5%)	63 (52.5%)		29 (52.7%)	66 (50.4%)	

It is important to inform the dentist about any past/current health conditions, allergies, treatments, or usage of medications										
Yes	163	86 (94.5%)	77 (81.1%)	0.005	55 (83.3%)	108 (90.0%)	0.186	45 (81.8%)	118 (90.1%)	0.118
No^*∗*^	23	5 (5.5%)	18 (18.9%)		11 (16.7%)	12 (10.0%)		10 (18.2%)	13 (9.9%)	

Some dental procedures need to be approved by the physician according to the patient health condition										
Yes	145	75 (82.4%)	70 (73.7%)	0.151	53 (80.3%)	92 (76.7%)	0.567	42 (76.4%)	103 (78.6%)	0.734
No^*∗*^	41	16 (17.6%)	25 (26.3%)		13 (19.7%)	28 (23.3%)		13 (23.6%)	28 (21.4%)	

Continuous usage of some medication can cause oral health problems like tooth decay and inflamed gums										
Yes	105	45 (49.5%)	60 (63.2%)	0.059	35 (53.0%)	70 (58.3%)	0.485	30 (54.5%)	75 (57.3%)	0.734
No^*∗*^	81	46 (50.5%)	35 (36.8%)		31 (47.0%)	50 (41.7%)		25 (45.5%)	56 (42.7%)	

Some individuals with special needs are at a higher risk of health problems related to poor oral health										
Yes	112	56 (61.5%)	56 (58.9%)	0.718	35 (53.0%)	77 (64.2%)	0.138	31 (56.4%)	81 (61.8%)	0.487
No^*∗*^	74	35 (38.5%)	39 (41.1%)		31 (47.0%)	43 (35.8%)		24 (43.6%)	50 (38.2%)	

It is important the dentist team communicate with other health providers to offer the best treatment for special needs patients										
Yes	161	83 (91.2%)	78 (82.1%)	0.069	53 (80.3%)	108 (90.0%)	0.064	46 (83.6%)	115 (87.8%)	0.449
No^*∗*^	25	8 (8.8%)	17 (17.9%)		13 (19.7%)	12 (10.0%)		9 (16.4%)	16 (12.2%)	

Bolded *p* value is significant at <0.05. ^*∗*^Includes responses from caregivers who do not know.

## Data Availability

The data supporting the findings of this study are included within the article and are available from the corresponding author upon request.
